# Engineering Fatty Acid Biosynthesis in Microalgae: Recent Progress and Perspectives

**DOI:** 10.3390/md22050216

**Published:** 2024-05-09

**Authors:** Yanhui Song, Fangzhong Wang, Lei Chen, Weiwen Zhang

**Affiliations:** 1 Laboratory of Synthetic Microbiology, School of Chemical Engineering and Technology, Tianjin University, Tianjin 300350, China; 15966211488@163.com (Y.S.); lchen@tju.edu.cn (L.C.); 2 Key Laboratory of Systems Bioengineering (Ministry of Education), Tianjin University, Tianjin 300350, China; 3 Center for Biosafety Research and Strategy, Tianjin University, Tianjin 300072, China

**Keywords:** microalgae, lipid production, fatty acid biosynthetic pathway, transcription factor, transcriptional regulation, genetic engineering

## Abstract

Microalgal lipids hold significant potential for the production of biodiesel and dietary supplements. To enhance their cost-effectiveness and commercial competitiveness, it is imperative to improve microalgal lipid productivity. Metabolic engineering that targets the key enzymes of the fatty acid synthesis pathway, along with transcription factor engineering, are effective strategies for improving lipid productivity in microalgae. This review provides a summary of the advancements made in the past 5 years in engineering the fatty acid biosynthetic pathway in eukaryotic microalgae. Furthermore, this review offers insights into transcriptional regulatory mechanisms and transcription factor engineering aimed at enhancing lipid production in eukaryotic microalgae. Finally, the review discusses the challenges and future perspectives associated with utilizing microalgae for the efficient production of lipids.

## 1. Introduction

Fossil fuel energy resources have gradually been exhausted, due to their non-renewable nature and extensive human consumption. The utilization of fossil fuels has triggered a series of environmental issues, primarily compromising air pollution and greenhouse gas emissions. Therefore, the urgency to drive energy transitions and to seek alternatives has become important [1]. Biodiesel, which consists of fatty acid methyl esters formed by the esterification of fatty acid with methanol, is a renewable and clean energy source, possessing fuel-like characteristics and an ignition performance similar to petrodiesel [2]. Currently, biodiesel is predominantly derived from edible vegetable oils sourced from crops such as soybeans and oil palm. However, crop-based biodiesel presents drawbacks in terms of a lengthier production cycle and creating competition for arable land with grain cultivation [3]. In contrast, microalgae have a rapid growth rate and a high lipid storage capacity [4]. Their photosynthetic efficiency, however, plays a crucial role in determining biodiesel yield. The efficiency of carbon dioxide fixation by microalgae, using solar energy, surpasses that of other terrestrial plants by 10–50 times [5]. Most microalgae can synthesize 20–50% lipids in their biomass, making them highly promising platforms for lipid production [6]. Microalgal oils contain both saturated fatty acids (SFAs) and long-chain polyunsaturated fatty acids (PUFAs). SFAs can be utilized for producing biodiesel and other oleochemical products, while PUFAs exhibit significant physiological functions. Long-chain PUFAs include docosahexaenoic acid (DHA, 22:6, ω-3), docosapentaenoic acid (DPA, 22:5, ω-3), and eicosapentaenoic acid (EPA, 20:5, ω-3). DHA is a major component of the human brain and retina, constituting up to 7.7% of total fatty acids (TFAs) in the human brain [7]. Previous studies have demonstrated that increased levels of EPA in the bloodstream significantly reduce the risk of developing coronary heart disease [8]. Moreover, EPA holds potential in attenuating oxidative stress, inflammation, cancer, hyperlipidemia, neurodegenerative diseases, and other ailments, exerting multiple beneficial effects on human health [9]. Research indicates that a daily intake of 250 mg EPA + DHA has been shown to provide primary prevention against cardiovascular diseases [10].

Microalgae serve as potential chassis for fatty acid production. Typical chassis include *Chlamydomonas reinhardtii*, *Nannochloropsis*, *Schizochytrium*, and diatoms. Microalgae, especially green algae, similarly to plants, utilize the classical fatty acid synthase (FAS) pathway for fatty acid synthesis. The precursor for fatty acid synthesis, acetyl-CoA, is converted by acetyl-CoA carboxylase (ACCase) and the FAS II complex to synthesize C16 or C18 FA. Subsequently, C16 and C18 FA are transported to the endoplasmic reticulum (ER) and further processed into triacylglycerols (TAGs). In the ER, acyl-CoA and glycerol-3-phosphate (G3P) are catalyzed sequentially by 3-phosphoglycerol acyltransferase (GPAT), lysophosphatidic acid acyltransferase (LPAAT), phosphatidic acid phosphatase (PAP), and diacylglycerol acyltransferase (DGAT), to generate TAG. While most TAG synthesis occurs via the DGAT reaction, there exists a non-acetyl-CoA-dependent pathway utilizing phosphatidylcholine (PC) as the acyl donor and diacylglycerol as the acceptor, catalyzed by phospholipids: diacylglycerol acyltransferases (PDAT). In addition to this, microalgae exhibit some differences in fatty acid synthesis. Firstly, certain microalgae, such as *Schizochytrium*, possess another PUFA synthesis pathway, catalyzed by a single polyketide synthase (PKS). This pathway involves a series of cycles composed of condensation, ketoreduction, dehydration, and enoylreduction reactions, catalyzed, respectively, by the ketoacyl-ACP synthase domain, ketoacyl-ACP reductase domain, dehydratase (DH) domain, and enoyl-ACP reductase domain of PKS, to synthesize PUFAs. Notably, a DH domain, similar to the FabA domain in *Escherichia coli* and called DH_FabA_, catalyzes the dehydration of 3-hydroxyacyl-ACP to form trans-2-enoyl-ACP, which is then isomerized to cis-3-enoyl-ACP or cis-2-enoyl-ACP, thus bypassing the enoyl reduction step in the cycle and preserving cis double bonds in the acyl chain [11]. Secondly, microalgae typically contain betaine lipids, such as diacylglycerol trimethylhomoserine (DGTS). Due to their structural similarity, DGTS is believed to substitute for PC. Moreover, in *Chlamydomonas*, DGTS is the only lipid present, lacking PC, which plays a vital role in plant lipid synthesis [12]. Thirdly, TAG assembly occurs not only in the ER but also possibly in the chloroplasts of microalgae such as *Chlamydomonas*. It has been confirmed that there is a partial Kennedy pathway from glycerol-3-phosphate to diacylglycerol in the chloroplast [6,12]. Fourthly, in some oil-producing microalgae, such as *Schizochytrium*, ATP citrate lyase (ACL) plays a key role in lipid accumulation, which is similar to other oil-producing microorganisms, such as *Yarrowia lipolytica* [13,14,15].

Compared with other oil-producing microbes, microalgae have relatively low fatty acid productivity. Therefore, improving their fatty acid content is a trending topic in this research area. This paper provides a comprehensive review of mechanisms and engineering strategies aimed at increasing oil content, with a focus on the following two main areas: one is the identification of key genes and associated engineering approaches involved in the fatty acid synthesis pathway, and the other is an exploration of transcriptional regulation mechanisms and engineering strategies for enhancing fatty acid synthesis. Additionally, the paper addresses current challenges and outlines future directions for engineering advancements aimed at achieving high microalgal fatty acid production.

## 2. Engineering the Fatty Acid Biosynthetic Pathway in Eukaryotic Microalgae

Enhancing fatty acid synthesis pathways is an important research direction. There are numerous excellent reviews available on this topic [16,17,18]. In the past, progress in the development of genetic tools and omics technologies has significantly enhanced our comprehension of lipid metabolism, thereby presenting opportunities for targeted metabolic engineering [19,20]. The first major breakthrough in microalgal biotechnology was the nuclear transformation of *C. reinhardtii* in 1990, and, since then, microalgae-based research has made tremendous advances in the field of lipid metabolic engineering over the past 30 years, involving the FAS pathway, the Kennedy pathway, the fatty acid elongation/desaturation pathway, the PKS pathway, lipid catabolism, and the bypass pathway [21,22].

**Figure 1:**
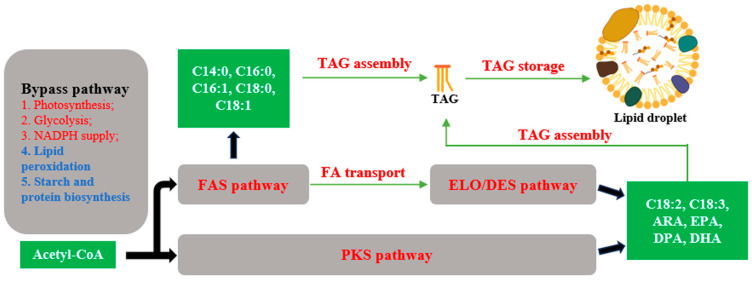
Strategies for engineering fatty acid biosynthetic pathway in eukaryotic microalgae. Pathways marked in red facilitate the biosynthesis of lipids. Pathways marked in blue repress the biosynthesis of lipids.

In this review, we aim to summarize the research progress made over the past five years (Figure 1). We will focus on three representative species of microalgae, namely *C. reinhardtii*, *Nannochloropsis* sp., and *Schizochytrium* sp. In recent years, *C. reinhardtii*, a non-oil-producing microalga, has been widely employed as a reference organism for research on TAG metabolism in algae, especially in the exploration of the key enzymes in fatty acid metabolic pathways, which will provide guidance for engineering photosynthetic oil-producing microalgae to increase oil production. The microalgae *Nannochloropsis* sp. is characterized by its high lipid content, which can reach up to 64.3% of its dry weight [23,24]. *Nannochloropsis* sp. has a high proportion of C18:1 and EPA in its fatty acid composition, making it well suited for the production of high-quality biodiesel and EPA [24]. *Schizochytrium* is a unicellular eukaryotic marine protist, belonging to the Thraustochytrids genera, and it is famous for its high lipid productivity and the PKS pathway. The algal oil of *Schizochytrium* is rich in DHA (35–40% of total lipids), is generally regarded as safe (GRAS), and has been industrially developed for human consumption [25].

### 2.1. Fatty Acid Synthase (FAS) Pathway and ω6-Elongase/Desaturase (ELO/DES) Pathway

Fatty acids (FAs) with different chain lengths have different application values. Wang et al. designed a cell factory model for FA chain length control at a unit chain-length resolution in *N. oceanica* and regulated the composition of C16, C18, or C20 fatty acids through NoTE1, an endogenous PKS type II acyl-ACP thioesterase, using C16:1-ACP and C18:1-ACP as substrates [26]. In addition, the overexpression of heterologous CpTE (*Cuphea palustris* acyl-ACP thioesterase) could increase the fatty acid composition of C8 and C10, and the chain length preference of the CpTE enzyme could be shifted to C12:0 using the rational design of its substrate-binding pocket [26].

*Nannochloropsis* is a genus of heterokont microalgae that are rich in EPA and may utilize the ω6-Elongase/Desaturase (ELO/DES) pathway for EPA biosynthesis [27]. Zhang et al. verified that the Δ9 fatty acid desaturase (FAD9) of *N. oceanica* prefers C18:0 as a substrate and found that Δ12, Δ5, and Δ17 fatty acid desaturases (FAD12/5/17) are the rate-limiting enzymes in its EPA biosynthesis [28]. The delta-9 stearoyl-CoA desaturase is the rate-limiting step in the lipid synthesis pathway of the oil-producing yeasts *Y. lipolytica* and *R*. *toruloides*. Whether FAD9 has the same function in the oil-producing microalgae *N. oceanica* requires further experimental characterization [29,30].

### 2.2. Polyketide Synthase (PKS) Pathway

The high DHA production of *Schizochytrium* is attributed to its unique and efficient PKS pathway. Among *Thraustochytrids*, some contain both PKS and ELO/DES pathways, but the PUFAs of *Schizochytrium* sp. are synthesized only via the PKS pathway, and the ELO/DES pathway of *Schizochytrium* sp. is incomplete [31,32]. The PKS pathway requires less NADPH and produces fewer intermediate fatty acids than the ELO/DES pathway [33]. The polyketide synthase gene cluster of *Schizochytrium* sp. consists of the following three subunits: ORFA, ORFB, and ORFC. Some domains included in the cluster have been functionally investigated. The dehydratase domain of ORFC (ORFC-DH_1,2_) plays a key role in PUFA synthesis and prefers to act on C16:0 [34,35]. The dehydratase domain of ORFA (ORFA-DH_3_) is beneficial to the biosynthesis of saturated fatty acids and tends to act on C14:0 and C15:0 [35]. The enoyl-ACP reductase domain of ORFB (ORFB-ER) also plays a key role in PUFA synthesis [36]. The enoyl-ACP reductase domain of ORFC (ORFC-ER) may be involved in the biosynthesis of saturated fatty acids (SFAs) and cell growth [34]. The ORFB chain length factor (CLF) may be one of the key genes involved in the elongation of the C20 to C22 PUFAs [37]. The algal oil of *Schizochytrium* is rich in DHA and DPA content and very low in EPA content because its PKS gene cluster is of the DHA/n-6 DPA type. However, Ma et al. found that EPA synthesized by *Schizochytrium* is also released via the PKS pathway. The control of EPA content in *Schizochytrium* is achieved by a cobalamin (VB_12_)-independent methionine synthase-like (MetE-like) complex, which can regulate the DHA/n-6 DPA-type PKS gene cluster [31]. When the MetE-like complex was activated (removing VB_12_ from the medium), the EPA content increased from 1.26% to 7.63%, a 6.06-fold increase from the inactivated condition, and EPA was more likely to be stored as TAG. However, it also led to lower biomass and lipid accumulation, because the transcription levels of ORFA, ORFB, and ORFC were downregulated [31].

### 2.3. Fatty Acid Transport

Fatty acid synthesis in photosynthetic microalgae is carried out in the plastid and then transported to the endoplasmic reticulum for TAG assembly. Therefore, the genetic reconstruction of fatty acid transport-related genes can help to increase the fatty acid content of *C. reinhardtii*. Joseph et al. found that the overexpression of C18:1-ACP-specific thioesterase could maintain a high rate of de novo FA biosynthesis. This phenomenon can be explained by the following two aspects. Firstly, after 24 h of N deprivation, the FA and TAG biosynthesis were strongly upregulated, and C18:1-ACP had a proportional increase with the time course, which indicates that C18:1-ACP may be a feedback inhibitor of ACCase [38]. Secondly, C18:1 is probably the main FA exported by the plastid [39]. Acyl-CoA is an important metabolic intermediate, which can mediate de novo acyl lipid synthesis, membrane lipid remodeling, and FA β-oxidation in peroxisomes. Thus, ATP-dependent long-chain acyl-CoA synthetases (LACS) play a central role in acyl lipid metabolism [40]. Bai et al. found that CrLACS1, CrLACS2 (ER localization, with functional overlap), and CrLACS3 (peroxisomal localization) were involved in the adjustment of lipid metabolism during nitrogen deprivation and nitrogen replenishment, respectively. Under the N deprivation condition, the inhibition of CrLACS1 or CrLACS2 resulted in a 50% reduction in TAG content but an increase in chloroplast lipid content. In contrast, the inhibition of CrLACS3 severely impaired TAG mobilization and cell growth during nitrogen replenishment [40]. The effects of the key enzymes of lipid metabolism in *C. reinhardtii* on lipid synthesis were mostly revealed under the nitrogen deficiency condition, such as LACS [40] and LPAAT [41], but nitrogen deficiency led to impaired biomass. Chen et al. found that simultaneous overexpression of the chlorophyll-localized FA exporters FAX1 and FAX2 and of the ER-localized FA transporter ABCA2 enhanced the accumulation of TAG, total fatty acids, and membrane lipids in *C. reinhardtii* under non-stress conditions, as well as facilitating the flow of PUFA from membrane lipids to TAG. In-depth analysis revealed that the expression of the gene that encodes the monogalactosyldiacylglycerol (MGDG)-specific lipase plastid galactoglycerolipid degradation 1 (PGD1) and genes that are directly involved in TAG synthesis, such as PDAT1 and DGTT1 (a type of diacylglycerol acyltransferase), were upregulated in the FAX1/FAX2/ABCA2-OE strains [42].

### 2.4. Triacylglycerol (TAG) Assembly

The storage of FA in TAG can provide a strong traction force for fatty acid biosynthesis. Moreover, from the perspective of biorefining, TAG is superior to polar membrane lipids in the downstream extraction of lipids from algal biomass [43]. G3P is an important precursor for triglyceride (TAG) synthesis under nutrient starvation conditions. Thomas et al. found that GPDH2 and GPDH3 (glycerin-3-phosphate dehydrogenase), which catalyze G3P synthesis in *C. reinhardtii*, are induced by nutrient starvation, and their transgenic lines brought significant changes in lipid concentration and composition to *C. reinhardtii* [44]. In addition, the overexpression of acyltransferases for TAG assembly, such as LPAAT, PDAT, and DGAT, has been shown to enhance lipid accumulation in *C. reinhardtii* [19,45]. Free fatty acids from membrane lipolysis can also be transferred to TAG for storage, and the key enzymes involved in the membrane lipid remodeling process are mainly some lipases and membrane lipid synthases. In algae and higher plants, MGDG accounts for more than 50% of total membrane lipids and undergoes membrane lipid remodeling under abiotic stress conditions, such as nitrogen starvation [46]. Li et al. found that PGD1 knockout mutants of *C. reinhardtii* acquired lower TAG levels than those of WT after N deprivation [47]. Lee et al. knocked down the expression of the gene that encodes MGDG synthase 1 (CrMGD1) and found that the MGDG content was reduced by 22%, and DGTS and TAG were increased by 1.39-fold and 5.40-fold, respectively [46]. Analyzing the reason for this phenomenon, it was found that, in addition to membrane lipid remodeling, increased DGTS levels induced endoplasmic reticulum stress, all of which contributed to the improvement in TAG accumulation and lipid properties of biodiesel feedstocks [46].

### 2.5. Triacylglycerol (TAG) Storage

Synthesized TAG is stored in lipid droplets (LDs). LDs are enveloped by a phospholipid monolayer with embedded lipid droplet proteins, which are deposited on the LD membrane [48]. Mass spectrometry analysis showed that the major lipid droplet protein (MLDP) was a major protein in lipid droplets, and the inhibition of the expression of the MLDP-encoding gene, using RNAi, led to an increase in lipid droplet size, but no changes in triacylglycerol content or metabolism were observed [49]. Lee et al. found that Delayed in TAG Hydrolysis 1 (DTH1) localizes to the LD, binds with phosphatidylethanolamine (the major phospholipid covering the surface of the LD), and contributes to the LD disappearance and TAG degradation after nitrogen restoration, which can provide carbon and energy for cellular growth. *dth1-1* mutants showed growth defects and a delay in TAG remobilization after N restoration [50].

### 2.6. Bypass Pathways

The enzymes in this section are not directly involved in lipid biosynthesis but affect lipid accumulation. *Schizochytrium* is considered an excellent strain for DHA production, but the DHA production is limited by the availability of NADPH. Feng et al. found that overexpression of G6PD in *Schizochytrium* increased the availability of NADPH, which ultimately led to an increase in lipid accumulation and DHA production [51]. The lipid productivity of photosynthetic microalgae is lower than heterotrophic oil-producing microorganisms, due to slower growth caused by photosynthetic autotrophy. The high efficiency of algal photosynthesis depends on its carbon concentration mechanism (CCM), which contributes to CO_2_ fixation [45]. Jeon et al. found that the overexpression of the endogenous NADP malic enzyme (NsME1) increased CO_2_ supply through CCM and increased the NADPH/NADP ratio in plastids, which increased the carbon source and reducing power supply for lipid synthesis, and finally improved the lipid and fatty acid methyl ester (FAME) production of *N. salina* without affecting growth [45].

The biosynthesis of starch, fatty acids, and proteins has competition in carbon fluxes. Therefore, the inhibition of competing pathways could improve fatty acid synthesis. Lee et al. proposed fructose-1,6-bisphosphate aldolase (CrFBA1) as a key regulatory gene of carbon metabolism in *C. reinhardtii*. In mixotrophic and nitrogen-depleted conditions, the overexpression of CrFBA1 increased the fatty acid content and decreased the starch content of *C. reinhardtii* [52]. RT-qPCR showed that CrFBA1 overexpression upregulated the exogenous acetate assimilation and glycolysis pathways, and the starch biosynthesis pathway was inhibited, which ultimately led to the enhanced conversion of starch to fatty acids [52]. Phosphoenolpyruvate (PEP) is an important branching point for carbon fluxes to enable protein synthesis or fatty acid synthesis. Phosphoenolpyruvate carboxylase (PEPC) catalyzes a large carbon flux into the TCA cycle and ultimately leads to protein synthesis. PEP is catalyzed sequentially by a pyruvate kinase and pyruvate dehydrogenase complex to synthesize acetyl-CoA, which drives carbon flux flow towards fatty acid biosynthesis [53]. Kao et al. used CRISPRi to transcriptionally silence the PEPC-encoding genes, which ultimately enhanced lipid production in *C. reinhardtii* [53].

During microbial fermentation for the production of lipids and polyunsaturated fatty acids, reactive oxygen species and reactive aldehydes generated can impair cell viability and cause lipid peroxidation. Lipid peroxidation is an important factor limiting DHA production in *Schizochytrium*, especially in the presence of large lipid accumulation [54]. Therefore, it is crucial to control the level of oxidizing substances in cells. Han et al. strongly promoted cell growth, lipid production, and DHA production by overexpressing thioredoxin reductase, aldehyde dehydrogenase, glutathione peroxidase, and glucose-6-phosphate dehydrogenase (ZWF) to enhance the oxidative stress defense pathway in *Schizochytrium* [55].

### 2.7. Combined Strategies to Improve Lipid Productivity

Single-gene modifications are suitable for studying gene functions, but their effect on lipid production is limited. With the gradual maturation of genetic manipulation systems in microalgae, a number of combinatorial strategies have emerged to improve lipid production through multi-gene editing. Han et al. performed a combinatorial genetic manipulation on the coding genes of ATP, citrate lyase (ACL), acetyl-CoA carboxylase (ACC), and peroxisome matrix protein (PEX10), which resulted in a 72.1% increase in DHA production in the engineered strain OACL-ACC/∆pex10. The lipid content of the engineered strain OACL-ACC/∆pex10 reached 82.2% in shake flasks, which is the highest lipid content reported to date in *Schizochytrium* [15]. Li et al. enhanced lipid and DHA accumulation in *Schizochytrium* using a combined strategy. Firstly, the competition between FAS pathway and PKS pathway by-products (DPA) was inhibited by the co-expression of phosphopantetheinyl transferase and ω-3 fatty acid desaturase. Then, lipid peroxidation was successfully prevented by a two-point addition of sesamol. The final lipid and DHA titers reached 92.5 g L^−1^ and 51.5 g L^−1^, respectively [56].

From the perspective of biorefining, TAG is more conducive to downstream extraction and purification than polar membrane lipids, so it is of great significance to improve the EPA content of TAG [43]. The push–pull strategy is a common method for metabolic engineering to increase yield. Liu et al. utilized a push–pull strategy to increase the EPA content in TAG in *N. oceanica*. EPA is transferred to TAG for storage and protection through the overexpression of heterologous CrDGTT1 with strong activity for EPA-CoA. This provides a strong traction for EPA synthesis. In addition to providing traction force, the additional overexpression of the elongase-encoding gene Δ0-ELO1, which acts on elongation from C16:0 to C18:0, further promoted the increase in EPA in TAG. Ultimately, the proportion of EPA in TAG and the content of TAG-derived EPA were 5.9-fold and 12.3-fold higher than those in the control strain, respectively [43].

## 3. Engineering Transcriptional Regulation of Fatty Acid Biosynthesis in Microalgae

The transcription factor (TF) serves as a powerful tool for modifying microbial metabolic pathways due to its unique advantage of multi-point regulation and dynamic regulation, enabling global, efficient, and specific modulation of target metabolic pathways. Transcription factor-based engineering strategies have been extensively employed and advanced in the field of metabolic engineering, encompassing the utilization of global transcription machinery engineering, the implementation of transcription factor-based biosensors, the application of quorum sensing systems for dynamic regulation, and the construction of artificial transcription factors [57]. In addition, in order to optimize the transcription regulation performance of TFs, much research has been devoted to modifying transcription factor regulation modes, such as the regulation of the specificity and location of transcription factor–DNA binding sites, multilevel modifications encompassing the protein structure and expression of the transcription factors themselves, and the coupling of transcription factors with other regulatory elements [57]. In the field of microalgae, due to the relative immaturity of the genetic operating system and the lack of understanding of the transcriptional regulatory mechanism, the research on transcription factor-based engineering strategies is limited to the overexpression, knockout, or knockdown of individual regulatory factors [21]. This review provides a comprehensive overview of transcription factors associated with lipid synthesis in microalgae, highlighting their potential as genetic engineering targets to enhance metabolic fluxes towards lipid biosynthesis. Paradigms of transcription factor engineering in microalgae are also summarized to generate Table 1.

### 3.1. Chlamydomonas reinhardtii

Nitrogen starvation increases lipid accumulation, but inhibits cell division and photosynthesis, which is not conducive to the large-scale cultivation of microalgae. However, elucidating the regulatory mechanisms underlying lipid accumulation in microalgae under nitrogen starvation conditions is a crucial step for achieving high lipid productivity. Transcriptomic studies of *C*. *reinhardtii* have suggested that TAG synthesis is a result of membrane remodeling via PDAT, PGD1, and putative lipases, as well as of *de novo* TAG synthesis catalyzed by DGAT [58]. Mahmoud et al. performed a combinatorial omics analysis of nitrogen-depleted *C*. *reinhardtii*, constructed a preliminary transcriptional regulatory network, and found a regulatory hub for lipid metabolism in *C. reinhardtii* [59]. During the early stage of nitrogen deprivation (BTS phase, before TAG synthesis, 0.5–4 h), the upregulation of the nitrogen starvation-specific response regulator NRR1 was observed, which correlates with increased transcription levels of DGTT1 and PLB2 (a putative phospholipase B-like protein). Subsequently, changes in the transcription levels of bHLH6 and bHLH9, GNAT7 and GNAT20, RWP1 and RWP10, bZIP3, HB2, TRAF8, and VARL12 show a positive correlation with the increased transcription levels of numerous lipases. Additionally, Tab2 and TAZ3 are associated with the biosynthesis of DGTS and control the rapid increase in DGTS content within 1 h after nitrogen depletion, followed by a steady decline. This correlation suggested the involvement of these early transcription factors in a large-scale regulatory network that controls membrane lipid remodeling under nitrogen-deficient conditions, and VARL12, HB2, bHLH9, and GNAT7 have been identified as BTS-specific regulatory hubs [59]. After the TAG synthesis phase (ATS, 6–24 h), several transcription factors play roles in membrane lipid remodeling and TAG biosynthesis. AP2-15, FHA10, SBP8, and MYBL13 have been identified as ATS-specific regulatory hubs. AP2-15 (a member of the AP2/EREBP family, CreWRI1-like) and FHA6 act by inhibiting the biosynthesis of sulfoquinovosyl diacylglycerol (SQDG) during nitrogen deprivation, thereby controlling the flow of fatty acids from photosynthetic membrane lipids to TAG. Furthermore, AP2-15 reprograms lipid composition by upregulating TAG biosynthesis via DGTT1. FHA10 and SBP8 function as inhibitors of TAG biosynthesis genes and activators of photosynthesis genes; they exhibit a negative correlation with the accumulation of TAG, DGTT1, and PGD1, while being positively correlated with many photosynthesis genes. MYBL13 shows a negative correlation with phosphatidylglycerol (PG) and two genes encoding PG synthase (PGPS1 and PGPS2); its upregulation after nitrogen depletion suggests its role in promoting lipid accumulation by regulating membrane lipid remodeling through the inhibition of PG biosynthesis [59].

The R2R3-MYB-type transcription factor CrMYB1 (referred to as CrMYBL13 by Gargouri et al., Cre01.g034350) is the primary regulatory factor for lipid accumulation under nitrogen depletion in *C*. *reinhardtii*, with the main function being the promotion of fatty acid transport from the chloroplasts to the endoplasmic reticulum [60]. Under growth conditions, it has also been observed to be closely associated with the expression of genes involved in de novo fatty acid synthesis and TAG assembly, resulting in a simultaneous increase in lipid, starch, protein, and biomass [62]. The *C. reinhardtii* MYB-related transcription factor ROC40 (rhythm of chloroplast 40) is dedicated to regulating lipid accumulation under nitrogen depletion, leading to significant but not extensive changes in oil content. One of the known regulatory mechanisms involves direct modulation of DGTT1. Given that ROC40 and CrMYB1 are both MYB TFs responsive to -N conditions and can alter the flux of fatty acids into TAG assembly, it is speculated that they may exhibit functional redundancy or interact with each other [59,60,63]. CrbZIP2 is also a transcription factor that is upregulated in response to nitrogen-deficient conditions, but it has multiple roles in the regulation of lipid and pigment metabolism in *C. reinhardtii* [64]. CrbZIP2 is a positive regulator of digalactosyl diglyceride (DGDG) degradation and a negative regulator of TAG degradation, carotenoid biosynthesis, and chlorophyll biosynthesis [64]. RNA-seq showed that the CrbZIP2 mutation resulted in the upregulation of several putative TAG lipases and the downregulation of several putative DGDG lipases. The carotenoid and chlorophyll biosynthetic pathways were also upregulated [64].

Under phosphorus starvation, both de novo TAG synthesis and membrane remodeling occur simultaneously. Membrane remodeling under P starvation is widely observed across all oxygenic photosynthetic organisms, ranging from cyanobacteria and phytoplankton to terrestrial plants. During this process, phospholipids are replaced by non-P glycolipids (such as SQDG) and/or betaine lipids to maintain membrane integrity throughout oxygenic photosynthetic organisms, releasing inorganic phosphate from the membrane to support other essential cellular processes [84,85]. The MYB-like transcription factor PSR1 is a global regulator involved in the regulation of the phosphorus starvation response. PSR1 is conserved in microalgae and higher plants [65,86,87]. The immediate homolog of PSR1 in higher plants is called AtPHR1. PSR1 may recognize the same P1BS element (GNATATNC) as PHR1 [65,88]. In addition to responding to P starvation stress, PSR1 is also upregulated in response to N and S depletion [60,66]. Under P starvation conditions, the starch accumulation in PSR1-overexpressing lines increases by 59%, while lipid accumulation decreases by 25%. However, in *psr1* mutants, both lipid and starch accumulations are inhibited. This is because there is an antagonistic relationship between starch and lipid synthesis. PSR1 has an upregulating effect on specific starch and lipid synthesis genes, but the intensity of the former is higher than the latter, resulting in a preferential increase in starch accumulation [65]. LRL1, which belongs to the R2R3-MYB family, is a lipid remodeling regulatory factor (Cre03.g197100) that responds to phosphorus starvation by simultaneously upregulating TAG biosynthesis and SQDG biosynthesis pathways [67]. A dataset from ALCOdb suggests that LRL1 is also upregulated under nitrogen starvation [89]. LRL1 and PSR1 exhibit different degrees of involvement in the phosphorus starvation response [67]. LRL1 is classified as a late-stage P starvation-induced TF, whereas PSR1 is an early stage P starvation-induced TF. Under phosphorus starvation conditions, both LRL1 and PSR1 can directly activate the expression of *sulfoquinovosyl diacylglycerol 2* (SQD2) in *C. reinhardtii*, and this trans-activation effect can be enhanced by two transcription factors—TTG1 and bHLH2 [67].

Appropriate salt stress conditions induce enhanced fatty acid accumulation in *C. reinhardtii* [90,91]. The growth and photosynthesis of *C. reinhardtii* are affected under the salt stress condition of 0.15 M NaCl, but lipid accumulation is increased [91]. Ji et al. identified six bZIP transcription factors (CrebZIP4, 5, 10, 11, 13, and 16) that exhibited differential expression under the salt stress condition of 0.15 M NaCl, and speculated about their potential involvement in the regulation of photosynthesis and lipid accumulation in *C. reinhardtii* under salt stress conditions [91]. Under the salt stress conditions caused by lower concentrations of NaCl (especially 0.1 M), *C. reinhardtii* could increase lipid production without decreasing its cell growth rate [92]. Larissa et al. identified 15 transcription factors and 7 transcriptional regulators involved in the adaptation to salt stress and the enhancement of lipid production in *C. reinhardtii* by nuclear proteomes. They highlighted five of these differentially expressed transcription factors, namely Cre02.g073650.t2.1, Cre02.g115250.t1.1, Cre10.g466250.t1.2 of the Top family, and Cre03.g176651.t1.1 and Cre03.g197350.t1.2 of the MYB family. Cre03.g176651.t1.1 and Cre03.g197350.t1.2 of the MYB family were previously identified as the histone H2A deubiquitinase MYSM1 and cell division cycle 5 (CDC5), respectively, and CrCDC5 has been shown to be a negative regulator of lipid accumulation in *C. reinhardtii* [92].

CrbZIP1 (Cre05.g238250) regulates lipid remodeling and activates the expression of many genes contributing to survival in *C. reinhardtii* under endoplasmic reticulum stress conditions. Initially localized on the ER membrane, CrbZIP1 undergoes transcript splicing mediated by upstream CrIRE1 during ER stress, resulting in the nuclear localization of the produced CrbZIP1. Yeast one-hybrid assays indicate that CrbZIP1 directly activates the expression of BTA1 and CrDES genes. BTA1 encodes a key enzyme involved in the biosynthesis of diacylglycerol trimethylhomoserine (DGTS) in the chloroplast outer membrane, while CrDES converts linoleic acid (18:2 Δ9,12) to α-linolenic acid (18:3, Δ5,9,12) through Δ5 desaturation. Under ER stress conditions, the crbzip1 mutant undergoes membrane lipid remodeling, with a significant increase in TAG content; notably, there is a sharp decrease in levels of α-linolenic acid [71].

Besides the stress response, other transcription factors associated with cell quiescence-related transcription programs, cell cycle regulation, or carbon flux have been identified as influencing lipid accumulation. Nutrient scarcity not only induces TAG accumulation but also triggers cell quiescence, affecting cellular growth, although this alteration is reversible upon nutrient replenishment. It has been observed that *C*. *reinhardtii* utilizes Compromised Hydrolysis of Triacylglycerols 7 (CHT7) to suppress quiescence-related transcription programs, facilitating rapid growth resumption. The loss of *cht7* leads to delayed growth and postponed TAG degradation upon nitrogen repletion, resulting in TAG accumulation [68]. *C*. *reinhardtii* CrCDC5 is a homolog of AtCDC5, which is a MYB DNA-binding protein known to be involved in the cell cycle of higher plants. Compared to the wild type (WT), the *crcdc5* mutant exhibits approximately a 166% increase in starch content and a 25% increase in oil content, but growth is inhibited. Further analysis reveals that the S/M phase is prolonged in the *crcdc5* mutant compared to the WT, indicating that the energy saved from slowed cell division is redirected towards the synthesis of reserve compounds such as starch and lipids [69]. The transcription factor CrDOF redirects carbon sources towards lipid synthesis, and its overexpression leads to increased total fatty acid content and cell dry weight, while reducing starch content [70,93,94].

Cullin assembles the cullin–RING E3 ubiquitin ligase (CUL) complex, the largest family of E3 ligases. Protein ubiquitination is a key mechanism for regulating physiological responses and cell development in eukaryotes [95]. In plants, CRL is involved in fatty acid metabolism. The CUL3-MATH-BTB/POZ E3 ligase complex interacts with the WRI1 transcription factor to alter fatty acid content in *Arabidopsis* seeds [96]. Luo et al. found that silencing the expression of CrCUL2 and CrCUL4, using RNAi, resulted in a decrease in biomass but an increase in lipid content by 20% and 28%, respectively [95]. By analogy with plants, CrCUL2 and CrCUL4 may play a negative regulatory role in lipid biosynthesis by degrading some key regulatory proteins.

### 3.2. Nannochloropsis

The Xu group predicted 30 transcription factors, belonging to 11 families, associated with lipid metabolism in *N*. *oceanica* IMET1. Among them, MYB-related (5), NF-YC (5), AP2 (4), and C3H (4) are predominant families. Additionally, a preliminary TF–transcription factor binding site (TF-TFBS) interaction network was established through prediction, including 11 TFs potentially involved in the transcriptional regulation of the TAG biosynthesis pathway. The TF bZIP was extensively characterized. Its binding sites were found in the promoter regions of acyl-CoA-binding protein (ACBP)-, 3-Ketoacyl-ACP synthase (KAS)-, LACS-, and LPAAT-encoding genes, and it upregulated the expression of these four genes [97]. A homolog of s259.g7362, named NsbZIP1, was identified in *N*. *salina* CCMP1776, whose overexpression simultaneously increased biomass and lipid productivity. After 12 days of cultivation, the biomass and lipid productivity of the overexpressing lines were higher than those of the wild type, under both normal culture conditions and nitrogen deficiency or high salt culture conditions [72]. Other TFs regulate multiple genes independently, such as s009.g891 (bZIP) activating the expression of genes encoding LPAAT and enoyl-ACP reductase, and s295.g8604 (AP2) inhibiting *PAP* and activating the expression of *LACS* and *KAS I*. Some TFs co-regulate certain genes, such as *LPAT* (regulated by three TFs collectively) and *DGAT-2A* (regulated by five TFs collectively) [97].

The overexpression of the transcription factor NobZIP1 in the microalga *N. oceanica* CCAP 849/10 (formerly CCMP1779) leads to a significant increase in lipid accumulation and secretion by 2-fold and 16.2-fold, respectively, without impairing growth and photosynthetic rates. The overexpression of NobZIP1 redirects energy and carbon precursors from protein and carbohydrate synthesis to lipid synthesis. ChIP-qPCR analysis reveals that key genes involved in lipid synthesis (*ACBP*, *KAS*, *LACS*, and *LPAAT*) were upregulated and cell wall polymer synthesis (*CPS* and *UGDH*) were downregulated by NobZIP1, thereby inducing excessive lipid production and secretion. Among these regulatory genes, UGDH is a critical enzyme in cell wall polysaccharide biosynthesis; thus, the downregulation of UGDH impairs the formation of cell wall polysaccharides, thereby playing a dual role in redirecting carbon flux towards lipid production and weakening cell walls [73].

A negative regulatory factor for lipid accumulation, ZnCys (Naga_100104g18), which is a homolog of the fungal Zn(II)_2_Cys_6_-encoding gene, was identified in *N. gaditana* CCMP1894. ZnCys (Naga_100104g18) was identified as a central regulator of lipid metabolism in *N. gaditana*. The deletion of ZnCys resulted in the impaired growth of mutants, although the total carbon-to-lipid allocation ratio increased from 20% to 40–55%. Nevertheless, under semi-continuous growth conditions, the lipid production of the ZnCys RNAi strain was increased; it produced almost twice the amount of lipids (~5.0 g m^−2^ d^−1^) as the wild type (~2.5 g m^−2^ d^−1^), with a minimal effect on growth [74]. NO09G01030, a homozygote of Naga_100104g18, was found in *N. oceanica*. HLM9 (a 3′ UTR-truncated mutant of NO09G01030) showed a 9% reduction in growth and a 40% increase in neutral lipid content, compared with the wild type [75].

An AP2-like transcription factor (NO06G03670) has been identified as one of three putative direct homologs of AtWRI1, a master regulator of seed oil accumulation in *Arabidopsis thaliana* [97]. HLM23, an insertion mutant of NO06G03670, increased by about 40% in neutral lipid content and improved photosynthetic performance without growth impairment [75]. Transcriptome analysis revealed that genes involved in plastid fatty acid synthesis, glycolysis, and the Calvin cycle were upregulated in HLM23 [75].

Overexpression of the transcription factor NsbHLH2 in *N. salina* CCMP1776 resulted in an increased growth rate and the accumulation of biomass. Under both normal and stress conditions (nitrogen deficiency and osmotic stress), the dry weight of NsbHLH2 transformants increased by 36–48% and over 20%, respectively, on the 8th day. Although there was minimal change in fatty acid methyl ester content, the FAME productivity was significantly higher than that of the wild type [76].

In *N*. *oceanica*, a blue light-inducible transcription factor, NobZIP77, was identified. Under nitrogen-sufficient conditions, NobZIP77 suppresses the expression of NoDGAT2B, whereas in nitrogen depletion conditions, it activates NoDGAT2B expression. Blue light reduces the binding of NobZIP77 to the promoter of NoDGAT2B, leading to the release of NoDGAT2B and increasing triacylglycerol production under nitrogen depletion [77].

### 3.3. Schizochytrium

The heterotrophic microalgae *Schizochytrium* accumulated large quantities of triacylglycerols rich in docosahexaenoic acid. The transcription regulation network related to fatty acids has been elucidated in recent years. The zinc finger protein LipR and the bZIP-type transcription factor FabR were both identified as negative regulators of lipid biosynthesis in *Schizochytrium*, exerting direct control over the transcription of genes encoding PKS synthase and FAS synthase [78,79]. The deletion of the *lipR* gene resulted in a significant increase of 33% in total lipids and 48% in DHA production [78]. Furthermore, FabR was found to be involved in the H_2_O_2_ stress response in *Schizochytrium*. The deletion of *fabR* led to enhanced growth rate, elevated DHA and lipid production (an increase of 30.1% and 46.5%, respectively), and improved antioxidant capacity compared to WT [79]. A RING finger domain-containing protein was identified in *Schizochytrium*, and it significantly increased fatty acid and β-carotene content, especially C14:0 [80].

### 3.4. Other Microalgae

In addition to these three typical microalgae, a number of transcription factors regulating lipid accumulation have been characterized in other microalgae. In *Phaeodactylum tricornutum*, the heat shock factor family (HSF) was the most representative of the TF family [98]. A heat shock transcription factor, PtHSF1, was found to be a positive addition for triacylglycerol synthesis through directly regulation with glycerol-2-phosphate acyltransferase [81]. In *Chlorella* sp., the overexpression of CvDOF improves lipid content but causes growth inhibition [82] and the overexpression of HSbZIP1 can enhance the lipid production of *Chlorella* sp. HS2 under heterotrophic conditions [83]. Under -N or TOR-inactivation conditions, the expression of four TFs (BRD1, HSF1, MYB3, and MYB4) increased, and then upregulated the expression of *LPAT1*, which was ultimately produced, leading to TAG accumulation in *Cyanidioschyzon merolae* [99]. The lipid content of *Dunaliella parva* was observed to increase from 25% to 40% under nitrogen limitation, and the transcription factor DpWRI1-like was upregulated in response to nitrogen limitation [100]. DpWRI1-like plays a global regulatory role in lipid accumulation. On the one hand, DpWRI1-like regulates target genes related to carbohydrate metabolism (including genes related to photosynthesis, glycolysis, the tricarboxylic acid cycle, and starch metabolism) to enhance the conversion of sugars to lipids. On the other hand, DpWRI1-like regulates target genes related to lipid metabolism (especially genes involved in fatty acid biosynthesis, such as *KAS*) to promote lipid production [101]. In addition, the transcription factors DpAP2 and WRKY were identified as associated with carotenoid synthesis in *Dunaliella* sp. [102,103].

Besides these experimentally characterized transcription factors, several transcription factors in microalgae have been identified by methods such as comparative transcriptomics and correlation analysis. Xing et al. initially identified the role of the AP2 superfamily and the R2R3-MYB family in the regulation of lipids and the response to temperature stress in the green alga *Auxenochlorella protothecoides* [104]. S. Thiriet-Rupert et al. identified the transcription factors NF-YB_2 and MYB-2R_14 as linked to photosynthesis and lipid synthesis in *Tisochrysis lutea* [105].

## 4. Conclusions and Future Perspectives

**Figure 2:**
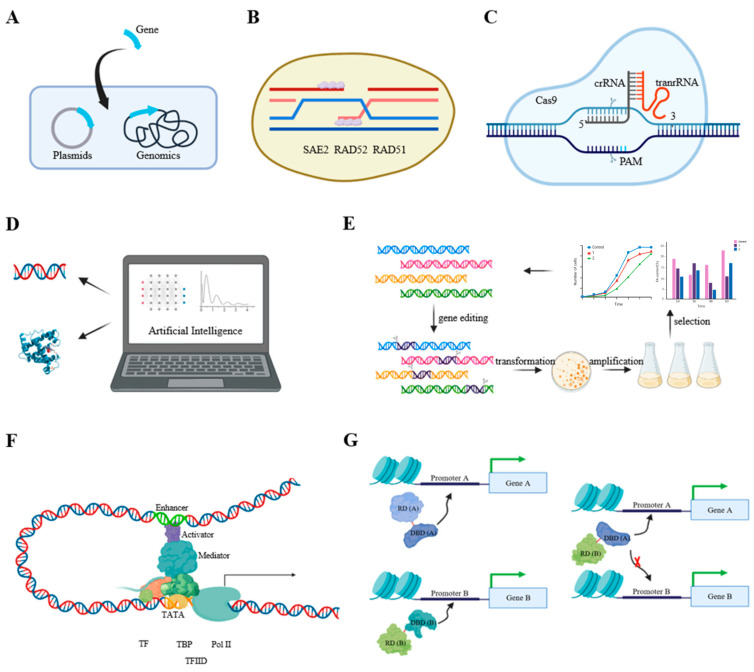
Further directions for increasing fatty acid production in microalgae. (**A**) Optimizing gene transformation efficiency; (**B**) increasing homologous recombination efficiency; (**C**) developing effective gene editing tools; (**D**) AI-assisted protein design; (**E**) genome-scale identification of new genes beneficial to lipid production; (**F**) in-depth exploration of transcriptional network; (**G**) construction of artificial transcriptional factors.

In this article, we have conducted a comprehensive review of recent advancements in the application of microalgal lipids for fatty acid production. Our focus primarily lies in the understanding and engineering of the fatty acid biosynthesis pathway, as well as the transcriptional regulation mechanisms governing this process. While significant progress has been achieved in this field, several key challenges for future research and development have been identified, which are summarized in Figure 2. These include the following: the development of efficient genetic manipulation tools; AI-assisted protein engineering of key genes; and the identification of relevant proteins beneficial to lipid production. A deep understanding of transcriptional networks of fatty acid biosynthesis and designs for new transcriptional strategies still remain to be addressed, in order to realize the industrial production of fatty acid.

### 4.1. Development of an Efficient Genetic Manipulation Toolbox for Oil-Producing Chassis

Although fatty acid synthesis regulation has been conducted in eukaryotic microalgae, their low biomass and lipid content limit their practical industrial applications. In order to rewrite the metabolic network, an efficient genetic toolbox is needed. However, oil-producing eukaryotic microalgae face the following challenges: firstly, the difficulty of gene transformation; secondly, the low efficiency of homologous integration; and thirdly, the lack of available genetic manipulation tools, when carrying out synthetic engineering reconstruction.

Genetic transformation methods include biolistic particle bombardment, glass bead agitation, electroporation, *Agrobacterium*-mediated transformation, silicon carbide whiskers, and spheroplast transformation [106,107]. Compared to other methods, electroporation is simple and less time-consuming. After optimizing the protocol, electroporation efficiency showed good progress. Recently, it was reported that a transformation method mediated by *Agrobacterium tumefaciens* AGL-1 was developed in *Schizochytrium* sp. HX-308, with a non-homologous recombination rate of 100% and a homologous integration efficiency of 30% [108]. However, due to significant differences among *Schizochytrium* species, it cannot be guaranteed that similar methods are applicable to other species. Optimizing gene transformation efficiency remains an important research direction for oil-producing microalgae.

A low homologous integration efficiency is also a major challenge for oil-producing chassis. An alternative method is to enhance homologous recombination efficiency, which has been successful in *Pichia pastoris*. It was found that the key homologous recombination gene RAD52 of *P. pastoris* was repressed, and the overexpression of exogenous genes such as ScSAE2, ScRAD52, and ScRAD51 from *S. cerevisiae*, as well as endogenous PpRAD52, could effectively increase homologous recombination efficiency [109]. In future studies, it is necessary to analyze and compare the efficiency of non-homologous recombination pathways and homologous recombination pathways in different oil-producing microalgae, identify key factors or genetic elements, and overexpress these, in order to increase the homologous recombination rate of oil-producing microalgae.

The lack of effective gene editing tools, such as CRISPR/Cas9/Cpf1/CRISPRi, which play an important role in gene deletion, insertion, point mutation, or repression, should receive attention. Due to toxicity, these tools are usually transformed into microalgal cells in the form of RNPs [110]. However, several challenges are encountered with these approaches in oil-producing chassis. Firstly, achieving homologous integration is difficult, and existing tools typically integrate into non-homologous loci, potentially leading to low-expression sites or the constitutive expression of sgRNA, which could result in toxicity or off-target effects. Secondly, the lack of autonomously replicating plasmids prevents seamless deletion, thereby limiting the ability to conduct multiple rounds of genetic manipulation. Moving forward, it is imperative to identify additional neutral integration sites, enhance the efficiency of homologous recombination, screen for more autonomously replicating sequences via genomic sequencing, and develop additional genetic manipulation tools to enable multiple rounds of genetic modification.

### 4.2. Artificial Intelligence-Assisted Protein Engineering of Key Proteins or Genome-Scale Identification of New Genes Beneficial to Lipid Production in Oil-Producing Chassis

As previously discussed, engineering strategies targeting the fatty acid biosynthesis pathway have yielded promising results in recent studies. However, several challenges still persist, including the following: the difficulty of customizing fatty acid profiles and the lack of new targets to enhance the fatty acid titer.

The fatty acid’s profile determines its related functions, and thus requires customization according to the researchers’ needs. Enzymes in the fatty acid synthesis pathway, such as 2-lysophosphatidic acid acyltransferase, diacylglycerol acyltransferase, and acyl-ACP thioesterase, exhibit strong substrate preferences [26,111,112]. The substrate preferences of enzymes determine the fatty acid profile. In oleaginous chassis, most studies focus on screening and characterizing enzymes with superior performances in nature. With the accumulation of protein databases, various machine learning or deep learning models have been established, greatly accelerating the protein engineering process. Even enzymes with fewer functional variants can be effectively constructed. For instance, UniRep, a model trained from 24 million sequences, can optimize the activity of a eukaryotic green fluorescent protein and a prokaryotic β-lactam hydrolyzing enzyme, using 24 or 96 mutant-labeled data as training sets [113]. Moreover, considering the physicochemical properties that determine protein structure and function can help in designing entirely new protein sequences with desired structures and functions. Novel protein design models like ProteinSolver can design entirely new protein sequences by considering the physicochemical properties that determine protein structure and function [114]. In future research, it is imperative to fully utilize these protein design models to design new fatty acid synthesis enzymes.

When the fatty acid pathway’s efficiency approaches theoretical limits, there is a need to develop new strategies. A further research direction involves identifying novel genetic modification targets that can enhance the microbial growth rate and the biomass while ensuring high fatty acid accumulation. Due to the multifactorial nature of growth influences, it is necessary to explore new targets at the whole-genome level. Relevant strategies include traditional and irrational mutagenesis screening to improve host growth, which has proved effective for various oil-producing microalgae. And multi-omics analysis under different growth rates to identify genes or metabolic pathways influencing growth can also be a valuable approach to improve growth. For oleaginous microalgae with incomplete genetic manipulation, it is a better strategy. Whole-genome perturbations, such as genome-scale target RNAi or genome-scale transcription activation, can be employed to discover new genetic modification targets in oil-producing microalgae. This strategy is applicable to chassis with well-established genetic manipulation capabilities, such as *Nannochloropsis*, of which genome-wide transcription activation and inhibition strategies have been established [115]. Genetic tools-assisted strain evolution is also an effective strategy. By employing the Tn5 transposon system for random insertion into any position of the genome or utilizing multi-omics automated gene editing methods for whole-genome random gene editing, a mutant library can be established. This library, coupled with selective pressures, facilitates the identification of genes and mutants directly associated with growth [116,117].

### 4.3. In-Depth Analysis of the Regulatory Network Governing Fatty Acid Synthesis and Designing Novel Engineering Strategies in Oil-Producing Microbes

The elucidation of transcriptional regulatory networks in oleaginous microbes is still in its infancy. Current studies focus on the discovery of new transcription factors, transcription factor binding sites, and the regulation of target genes by transcription factors. However, the understanding of relationships between other transcription factors or the initiation transcription complex remains unclear. In terms of transcriptional engineering, the current approaches mainly involve the overexpression or deletion/downregulation of single transcription factors, necessitating the development of novel engineering strategies.

Evidence suggests that the transcriptional mechanisms governing plant lipid synthesis hold significant relevance for oil-producing chassis. Firstly, the heterologous expression of plant-derived transcription factors has been shown to enhance lipid content in oil-producing microalgae without compromising growth. For instance, the overexpression of AtWRI1 and AtLEC1 in *N*. *salina* and *C*. *ellipsoidea*, respectively, resulted in a substantial increase in lipid content while maintaining growth [118,119]. Secondly, homologs of transcription factors involved in plant lipid regulation also exert similar influences on lipid accumulation in microalgae. For example, while AtMYB118 regulates lipid accumulation at the spatial level in *Arabidopsis* seeds, its homolog CrMYB1 in *C*. *reinhardtii* acts as a key regulator of lipid accumulation under nitrogen-depleted conditions [59,60,61]. WRI1 is a master regulator of plant seed oil accumulation, and homologs of WRI1 in *C. reinhardtii*, *Nannochloropsis*, and *Dunaliella parva* also play important roles in their respective lipid accumulation [59,75,101,120]. Thirdly, microalgae and plants, both being photosynthetic organisms, share similarities in the mechanisms regulating lipid accumulation. For example, in *Arabidopsis*, the overexpression of core clock regulators MYB TF CCA1 and LHY can increase seed TAG accumulation [121]. Similarly, in *C*. *reinhardtii*, the transcription factor ROC40 was initially identified as a key regulatory component of the circadian rhythm system, and later found to be involved in regulating lipid accumulation under nitrogen depletion conditions [63]. This suggested a connection between lipid metabolism and circadian rhythm regulation in both microalgae and plants. Future research could leverage insights from the transcriptional regulatory network of plant lipid synthesis to advance our understanding and engineering capabilities for lipid synthesis in oil-producing microorganisms.

Strategies for transcriptional engineering in oil-producing chassis organisms are still under development. Several feasible transcriptional engineering strategies include the following: firstly, the overexpression or knockout of a single transcription factor; secondly, the co-expression of transcription factors; thirdly, the modification of transcription factor binding sites in the target gene promoter region; and fourthly, designing synthetic transcription factors. The first two strategies have achieved good results in most oil-producing microorganisms [122]. The third strategy is less suitable for most oil-producing chassis, due to immature genetic manipulation platforms. Artificial transcription factors have been successful in various chassis; however, few studies have been reported for oil-producing chassis. Artificial transcription factors offer advantages, such as the ability to change the expression pattern of single target genes by adjusting activation or inhibition domains, and the ability to regulate differential expressions of multiple target genes by adjusting the binding strength between binding domains and DNA, leading to significant phenotypic changes [123,124]. After the establishment of a candidate strain library through engineering modifications, high-throughput screening methods are required. Flow cytometry combined with fluorescence-activated cell sorting has been used for screening high-yield fatty acid strains. A fluorescence-based flow cytometry screening method using Nile red has been established in oleaginous yeast to determine the relative lipid content of modified strains and to evaluate the synergistic effects of key genes [125]. The strategy of “artificial transcription factors with flow cytometry coupled with fluorescence-activated cell sorting” is a promising strategy for improving fatty acid production.

## Figures and Tables

**Table 1:** TF engineering for lipid synthesis in microalgae.

TF and TF Family	Species	Target or Regulatory Genes	Effects on Lipid Biosynthesis	Transcription Factor Engineering			Comments	References
				Strategy	Lipid	Growth		
NRR1 (the SQUAMOSA promoter-binding protein)	*C. reinhardtii*	Positively correlated with the expression of DGTT1 and PLB2.	Positive	Knockout	The TAG was reduced by 50% under N deprivation.	--	A TF in specific response to N starvation during the BTS phase.	[58,59]
CrMYB1 (R2R3-MYB)	*C. reinhardtii*	Indirectly activated the transcription of FAT1, FAX1, FAX2, LPAAT, ACC1, KAS1, LACS1 and DGTT2-DGTT5.	Positive	Knockout	Under N deprivation, the following occurred: (1) the TAG content was reduced by approx. 66%; (2) the ratio of TAG/TFA was also reduced; and (3) in FA composition, the PUFA content was increased.	Increased.	(1) Major regulators of lipid accumulation under nitrogen depletion in *C. reinhardtii*; (2) the scope of regulation involves *de novo* fatty acid synthesis, plastid–ER transportation, TAG assembly, and membrane lipid remodeling.	[60,61]
				Overexpression	Overexpression under standard growth conditions resulted in a synergistic increase in lipids, starch, proteins, and biomass.	Increased.		[62]
ROC40 (MYB-related)	*C. reinhardtii*	Directly activated the transcription of DGTT1.	Positive	Mutation	Total lipid content was no longer increased under N starvation, but the flux of FA conversion to TAG was significantly reduced by 3.82%.	--	A TF in specific response to N starvation.	[63]
CrbZIP2 (bZIP)	*C. reinhardtii*	Indirectly activated putative TAG lipases, carotenoid, and chlorophyll biosynthetic pathways; indirectly suppressed putative DGDG lipases.	Negative	Mutation	Under N deprivation, TAG content was reduced, and DGDG, carotenoid, and chlorophyll content were increased.	Had no effect.	(1) In response to the N starvation; (2) simultaneously regulated lipid and pigment metabolism.	[64]
PSR1 (MYB-like)	*C. reinhardtii*	--	Positive	Overexpression	Lipid accumulation was reduced by 25% under P starvation.	Under -P conditions, there was no difference; under +P conditions, growth increased.	Early P starvation-induced TF.	[65]
				Knockout	Under -P conditions, lipid accumulation was reduced by more than 50%.	Under -P conditions, there was a growth defect; under +P conditions, there was no difference.		
				Overexpression	TAG content was increased under eutrophic conditions.	There was no difference under eutrophic conditions.		[66]
LRL1 (R2R3-MYB)	*C. reinhardtii*	Directly activated the transcription of SQD2 and indirectly activated the transcription of GPDH, DGTT1, MLDP, SQD1, and RCC1.	Positive	Knockout	Under -P conditions, lipid accumulation was reduced; compositionally, the molar percentage of SQDG was reduced.	Slower growth.	Late P-starvation-induced TF.	[67]
CHT7 (CXC domain–DNA binding protein)	*C. reinhardtii*	--	Negative	Knockout	Caused a delay in TAG degradation after N replenishment.	Caused a delay in growth degradation after N replenishment.	Shut down quiescence-associated transcriptional programs for the rapid reestablishment of growth.	[68]
CrCDC5 (MYB-related)	*C. reinhardtii*	--	Negative	Knockout	Caused a 25% increase in lipid content.	Suppressed growth.	Influenced the cell cycle.	[69]
CrDOF (Dof)	*C. reinhardtii*	Indirectly activated the transcription of BCC1, FAT1, SQD1, MGD1, DGD1, and PGP1 and indirectly suppressed the transcription of ACP1, ACS1, CIS1, and SQD2.	Positive	Overexpression	Total fatty acid content was increased by 23.24%; compositionally, UFA content increased significantly.	Increased.	Redirection of carbon sources.	[70]
CrbZIP1 (bZIP)	*C. reinhardtii*	Directly activated the transcription of BTA1 and CrDES.	Negative	Knockout	With 1 μg/mL clindamycin treatment, TAG content was increased; in composition, the level of pinolenic acid was drastically reduced.	--	Membrane lipid (DGTS) remodeling.	[71]
NsbZIP1 (bZIP)	*N. salina*	Indirectly activated the transcription of ACBP, KAS, LACS, and LPAAT.	Positive	Overexpression	FAME productivity was increased.	Biomass productivity was increased.	--	[72]
NobZIP1 (bZIP)	*N. oceanica*	Directly activate the transcription of ACBP, KAS, LACS, LPAAT, CPS, and UGDH.	Positive	Overexpression	Lipid accumulation and lipid secretion were increased by 1-fold and 16.2-fold, respectively.	Had no effect.	Redirection of carbon flow and increased lipid secretion.	[73]
ZnCys (Zn(II)_2_Cys_6_)	*N. gaditana*	--	Negative	Silenced by RNAi.	FAME productivity was twice as high as WT under semi-continuous growth conditions.	Nearly had no effect.	Redirection of carbon flow.	[74]
NO06G03670 (AP2-like)	*N. oceanica*	Indirectly suppressed the transcription of LACS and the FAS pathway.	Negative	Mutation	Neutral lipid content was increased by about 40%, and photosynthesis was improved.	Nearly had no effect.	Putative orthologs of AtWRI1.	[75]
NsbHLH2 (bHLH)	*N. salina*	--	Positive	Overexpression	Lipid content was unchanged, but FAME productivity was significantly higher than WT.	Growth rate was accelerated and biomass was increased.	Increased biomass resulted in increased FAME productivity.	[76]
NobZIP77 (bZIP)	*N. oceanica*	Directly suppressed the transcription of NoDGAT-2B.	Negative	Knockout	TAG productivity was increased by nearly 2-fold.	Had no effect.	Suppression was mitigated by nitrogen deficiency and blue light.	[77]
LipR (zinc finger)	*Schizochytrium* sp.	Directly suppress the transcription of pks, fas, acc, acl, ampD, fabD, mae, zwf, and dga1.	Negative	Knockout	The yields of total lipids and DHA were increased by 33% and 48%, respectively.	Had no effect	Directly suppressed genes encoding PUFA synthase and FAS synthase.	[78]
FabR (bZIP)	*Schizochytrium* sp.	Directly suppressed the transcription of acl, fas, and pks.	Negative	Knockout	The yields of total lipids and DHA were increased by 30.1% and 46.5%, respectively.	Had no effect	DNA binding activity was regulated in a redox-dependent manner.	[79]
S-R (A ring finger domain-containing protein)	*Schizochytrium* sp.	--	Positive	Overexpression	TFA content was increased by 29–36%.	Had no effect	Simultaneously increased fatty acid and β-carotene content.	[80]
PtHSF1 (HSF)	*P. tricornutum*	Directly activated the transcription of *GPAT3* and *DXS*.	Positive	Overexpression	TAG content was increased.	--	Simultaneous regulation of TAG and fucoxanthin synthesis.	[81]
CvDOF (Dof)	*C. vulgaris*	--	Positive	Overexpression	Under -P conditions, neutral lipid content per cell was approximately 1.5-fold higher.	Inhibition of growth.	Sacrificing growth to increase lipid and protein synthesis.	[82]
HSbZIP1 (bZIP)	*Chlorella* sp. *HS2*	Indirectly activated the transcription of ACC1, KCS4, and KCS11.	Positive	Overexpression	Under heterotrophic conditions, FAME yields were 74% and 113% higher.	Increased dry cell weight.	Simultaneous increase in growth and lipid content.	[83]

## Abbreviations

**Full Name**	**Abbreviation**
saturated fatty acid	SFA
polyunsaturated fatty acid	PUFA
docosahexaenoic acid	DHA
docosapentaenoic acid	DPA
eicosapentaenoic acid	EPA
total fatty acid	TFA
fatty acid synthase	FAS
acetyl-CoA carboxylase	ACCase, ACC
endoplasmic reticulum	ER
triacylglycerols	TAG
glycerol-3-phosphate	G3P
3-phosphoglycerol acyltransferase	GPAT
lysophosphatidic acid acyltransferase	LPAAT
phosphatidic acid phosphatase	PAP
diacylglycerol acyltransferase	DGAT, DGTT, DGA1
phosphatidylcholine	PC
phospholipids: diacylglycerol acyltransferases	PDAT
polyketide synthase	PKS
dehydratase domain of PKS	DH
diacylglycerol trimethylhomoserine	DGTS
ATP citrate lyase	ACL
fatty acid	FA
thioesterase	TE
fatty acid desaturase	FAD
elongase/desaturase	ELO/DES
chain length factor	CLF
three subunits of the PKS gene cluster	ORFA, ORFB and ORFC
methionine synthase-like complex	MetE-like complex
long-chain acyl-CoA synthetases	LACS
FA exporter	FAX1, FAX2
FA transporter	ABCA2
plastid galactoglycerolipid degradation 1	PGD1
glycerin-3-phosphate dehydrogenase	GPDH
regulator of chromosome condensation 1	RCC1
monogalactosyldiacylglycerol	MGDG
MGDG synthase 1	MGD1
lipid droplet	LD
major lipid droplet protein	MLDP
Delayed in TAG Hydrolysis 1	DTH1
carbon concentration mechanism	CCM
fatty acid methyl ester	FAME
NADP-dependent malic enzyme	ME, MAE
fructose-1,6-bisphosphate aldolase	FBA
phosphoenolpyruvate	PEP
phosphoenolpyruvate carboxylase	PEPC
glucose-6-phosphate dehydrogenase	ZWF
peroxisome matrix protein	PEX10
transcription factor	TF
before TAG synthesis phase	BTS
after TAG synthesis phase	ATS
a putative phospholipase B-like protein	PLB2
sulfoquinovosyl diacylglycerol	SQDG
phosphatidylglycerol	PG
PG synthase	PGPS1, PGPS2
digalactosyl diglyceride	DGDG
cullin-RING E3 ubiquitin ligase	CUL
acetyl-CoA biotin carboxyl carrier 1	BCC1
fatty acyl–acyl carrier protein thioesterase	FAT1
UDP-sulfoquinovose synthase	SQD1
digalactosyldiacylglycerol synthase	DGD1
phosphatidyl glycerophosphate synthase	PGP1
acyl-carrier protein	ACP1
acetyl-CoA synthetase	ACS1
citrate synthase	CIS1
sulfolipid synthase	SQD2
acyl-CoA-binding proteins	ACBP
3-Ketoacyl-ACP synthase	KAS
putative capsular polysaccharide synthesis	CPS
UDP-glucose dehydrogenase	UGDH
AMP deaminase	AMPD
malonyl-CoA/acyl carrier protein malonyltransferase	FABD
betaine lipid synthase 1	BTA1
1-deoxy-D-xylulose 5-phosphate synthase	DXS
β-Ketoacytl-CoA synthetase	KCS
